# Implication of Platelets in Immuno-Thrombosis and Thrombo-Inflammation

**DOI:** 10.3389/fcvm.2022.863846

**Published:** 2022-03-25

**Authors:** Younes Zaid, Yahye Merhi

**Affiliations:** ^1^Laboratory of Materials, Nanotechnology and Environment, Faculty of Sciences, Mohammed V University in Rabat, Rabat, Morocco; ^2^Immunology and Biodiversity Laboratory, Department of Biology, Faculty of Sciences, Hassan II University, Casablanca, Morocco; ^3^Laboratory of Thrombosis and Hemostasis, Montreal Heart Institute, Research Center, The Université de Montréal, Montreal, QC, Canada

**Keywords:** platelets, immuno-thrombosis, thrombo-inflammation, NETosis, extracellular vesicles (EVs)

## Abstract

In addition to their well-described hemostatic function, platelets are active participants in innate and adaptive immunity. Inflammation and immunity are closely related to changes in platelet reactions and enhanced platelet function in thrombo-inflammation, as well as in microbial and virus infections. A platelet’s immune function is incompletely understood, but an important balance exists between its protective and pathogenic responses and its thrombotic and inflammatory functions. As the mediator of vascular homeostasis, platelets interact with neutrophils, bacteria and virus by expressing specific receptors and releasing granules, transferring RNA, and secreting mitochondria, which controls hemostasis and thrombosis, infection, and innate and adaptive immunity. This review focuses on the involvement of platelets during immuno-thrombosis and thrombo-inflammation.

## Introduction

Platelets are small (2–4 μm in diameter) anucleated cells derived from their megakaryocyte’s precursors with 7–10 days lifespan. Nearly one trillion platelets sentinel the blood vessels to monitor and preserve the integrity of the vasculature. There is no nucleus in platelets, but they are prepacked with proteins and various forms of RNA from their precursor cells. When damage to blood vessels occurs, it triggers the formation of a thrombus to stop bleeding (1). The discoid shape changes to a spherical one, resulting in long filopodia that facilitate adhesion. In order for platelets to function, they must involve an array of adhesive and activation receptors, secreted granule reservoirs, and dynamic cytoskeletal proteins (2). Extracellular vesicles (microparticles) can also play a role in the formation of thrombus mediated by platelets, as they provide anionic phospholipids that aid in the coagulation process (3). The role of platelets is not restricted to the hemostatic/thrombotic response, but platelets play a crucial role in inflammatory and immune responses (4–8). Indeed, not only do platelets express an array of molecules serving wound repair, but they also bear immune and inflammatory molecules such as interleukin IL1 (9), and an array of receptors including toll-like-receptors (TLRs), CD154, or CD40L (4), Fc receptor for IgG (FcγRIIA) (10), IgA (FcαRI) and IgE (FcεRI) (11, 12).

In response to harmful pathogens, platelets contribute to the immune system either directly, by producing cytokines and antimicrobial peptides, or indirectly, through interactions with neutrophils, monocytes, lymphocytes, and other cells (13, 14). Immuno-thrombosis may negatively affect hemostatic and immunological processes during a bacterial infection, resulting in adverse clinical outcomes (15). In this review, we will focus on the role of platelets in immuno-thrombosis and thrombo-inflammation.

## Platelet-Associated Immunopathology: Immuno-Thrombosis and Thrombo-Inflammation

### Mechanisms of Neutrophil Extracellular Trap-Induced Thrombosis

It is unsurprising that platelet-neutrophil interactions are greatly increased during inflammatory responses (16–19). For the most part, soluble mediators initiate these interactions, which directly activate these cells. Platelets and neutrophils co-incubated with septic patient plasma induced platelet adhesion to neutrophils mediated by the TLR4 receptor (20, 21).

Neutrophils are the most abundant subset of leukocytes in arterial thrombi from patients with myocardial infarction (22). Activated neutrophils express adhesion molecules belonging to the selectin and integrin families promoting platelets and the endothelium binding (23). As well, activated platelets express adhesion molecules on their surface membrane, such as P-selectin that mediates binding of platelets to its main receptor on neutrophils, P-selectin-glycoprotein-ligand-1 or PSGL-1 (24). Indeed, neutrophils and activated platelets can recruit each other to inflamed or injured tissues, thereby causing thrombo-inflammation (25). In this regard, we found that platelets can modulate neutrophil adhesion to the injured arterial wall and that both elements influence the degree of post-injury vasoconstriction in *in vivo* porcine model involving arterial injury by angioplasty (26). More recently, we have revealed that platelet activation and binding to neutrophils enhance the secretion of platelet MMP-2 via an adhesive interaction between P-selectin and PSGL-1, which contributes to increase platelet-neutrophil aggregation (27). Inhibition of platelet-leukocyte binding, using a recombinant PSGL-1 reduced restenosis (28) and prevented in in-stent restenosis via reduction of thrombo-inflammatory reactions (29).

Neutrophil extracellular traps (NETs) are composed of DNA, histones, and antimicrobial peptides, and they are produced as part of an antimicrobial mechanism, which is affected by immune/immune-related cells during NETosis (30). Indeed, NETosis appears to be associated with many inflammatory disorders, including infections, cancers, endothelial dysfunction, atherosclerosis, thrombosis, and ischemia (31, 32).

Neutrophil extracellular traps contribute to thrombosis through direct and indirect mechanisms. Although the vast majority of studies use NET components rather than intact NETs, the role of intact versus NET component in activating coagulation is controversial (33). In addition to their ability to promote thrombin formation, NETs were also known for providing a scaffold for pro-coagulant molecules such as VWF, fibrinogen, FXII, and tissue factor, as well as pro-coagulant extracellular vesicles TF-bearing EVs for instance (34–38).

Platelets can induce dysregulation of NET, resulting in tissue damage, hypercoagulability, and thrombosis (34). In addition, NETosis is well documented in its role in the pathogenesis of sepsis and ARDS, causing vascular tissue damage and spreading microthrombi that eventually cause multiorgan failure and death (39, 40).

The studies on NETosis provide growing evidence that NETosis is connected to inflammation, atherosclerosis, and atherothrombosis, as well as poor prognoses of ischemic/reperfusion injuries (41).

Mouse studies have examined the effects of NETosis and platelet aggregation on outcome after ischemia/reperfusion (42). Then, Cf-DNAs trigger DNA-platelet and DNA-platelet-granulocyte colonization. This form of NET and platelet aggravation leads to NETosis.

**FIGURE 1:**
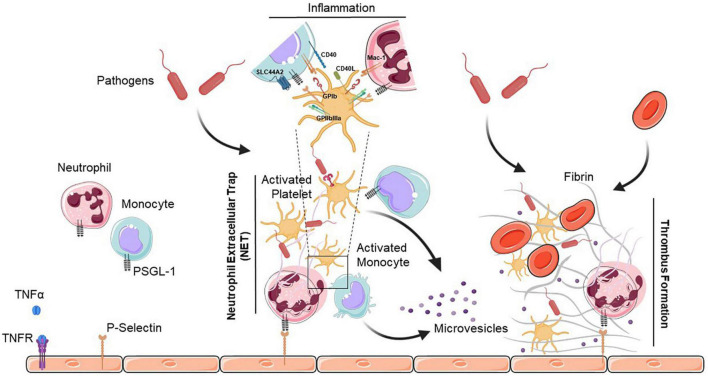
Neutrophil extracellular trap (NET)-mediated platelet thrombosis. In the presence of pathogens, TNFα binds to its receptor (TNFR) to initiate diverse cellular responses, and platelets, neutrophils and monocytes collaborate to form NETs that are highly thrombotic and resistant to tissue plasminogen activator-mediated fibrinolysis. NETs also help to recruit platelets which support the immuno-thrombotic process by promoting fibrin generation along with endothelial cells. Activated platelets release large amounts of pro-inflammatory cytokines in platelet extracellular vesicles. Interactions between platelets and neutrophils or monocytes occur via direct contact between cell surface receptors, or by binding of secreted ligands. PSGL-1, P-selectin glycoprotein ligand-1; TNFα, tumor necrosis factor alpha; TNFR, tumor necrosis factor receptor; GPIb, glycoprotein Ib; SLC44A2, solute carrier family 44 member 2; CD40L, cluster of differentiation 40 lignad; CD40, cluster of differentiation 40; Mac-1, macrophage-1 antigen; GPIIbIIIa, glycoprotein IIb/IIIa.

There are no doubts that NETs play a significant role in thrombosis and hemostasis (Figure 1). Both arterial and venous thrombosis are affected by NETs, and NETs are implicated in stenosis that regulate thrombosis in different ways (43, 44). Additionally, preventing the formation of NETs can reduce thrombogenicity, which could be useful in preventing thrombosis (44). A thorough understanding of how new therapeutic options targeting NETs affect thrombosis will require both preclinical and clinical trials.

### Platelet Extracellular Vesicles

In view of the critical role for platelets in thrombosis, studying platelet function may provide novel biomarkers for arterial thrombosis (45). There are numerous platelet function tests available in clinical practice, but most commonly, aggregometry-based tests are carried out (46). The aggregometry method, however, provides information on platelet functionality in the presence of exogenous and soluble agonists, which does not represent *in vivo* platelet activation. Thus, so far results from platelet function tests *in vitro* have limited value as biomarkers of arterial thrombosis.

A biomolecule or metabolite associated with activated platelets may be able to provide information about arterial thrombosis (47). Platelet-derived extracellular vesicles (PEV) are among these biomarkers, covering platelet microparticles/microvesicles and exosomes (48).

A number of benefits attributed to platelets are likely mediated by platelet-derived extracellular vesicles (PEVs), which are small vesicles released from activated platelets. Indeed, the release of platelet α-granules have been considered as small vesicles, as revealed by electron microscopic analyses (49). Also known as microparticles, this release of vesicles from the platelet plasma membrane occurs as a result of the extrusion of the platelet cytomembrane structures (50). Later studies using electron microscopy confirmed the presence of the two types of PEVs: small vesicles with diameters of 80–200 nm, and larger vesicles with diameters of 400–600 nm, which retained procoagulant activity mediated by factor V-like activity and tissue factor (51).

Platelet-derived extracellular vesicle can serve as biomarkers for autoimmune diseases, cancer, cardiovascular diseases, and infectious diseases (52–55). Rheumatoid arthritis patients have PEV detected in their synovial fluid (56), and increased levels of circulating PEV correlate with disease activity (57). Additionally, in murine models of atherosclerosis and autoimmune arthritis, PEV concentrations have been found to be increased in lymph (52, 58). As a result of systemic lupus erythematosus (SLE), PEV levels in blood can be increased. Higher levels of PEV have been associated with declining kidney function (59). Recently, an increase in PEV circulating in blood of COVID-19 patients has been observed (55, 60, 61).

Aside from transporting and producing inflammation-promoting mediators such as prostaglandins and leukotrienes, the PEV is also the transporter and producer of many lipid mediators (62).

## Receptors in Platelet–Immune Cell Interactions

Platelets enhance leukocyte recruitment by expressing an arsenal of complement system receptors including cC1qR, gC1qR, C3aR, and C5aR, and storing complement proteins and regulators such as C3 and factor H. Platelets stimulate the classical and alternative pathways of complement, causing the accumulation of opsonin C3b as well as the release of anaphylatoxins C3a and C5a, which chemoattract innate immune cells (63, 64).

Among innate immune system receptors, TLRs are pattern recognition receptors that link various pathogen-shared molecules, including lipopolysaccharide (LPS), lipoproteins, and other bacterial wall constituents (65). As a consequence of expressing TLRs involved in the innate immunity response, platelets may contribute to the immune response and to infections by secreting a number of inflammatory mediators and pro-inflammatory factors (5, 66, 67).

Recent studies found significantly elevated levels of CXCL1, CXCL8, CXCL12, CCL2, CCL3, CCL5, EGF, VEGF, and PDGF-AB/BB in plasma and BAL fluid of patients with severe COVID-19, all of which can be found in platelet granules, indicating that platelets may play a role in hyperinflammation observed in patients with ARDS (68, 69). These factors mediate the inflammatory response by acting synergistically, through both autocrine and paracrine pathways.

There is a wealth of evidence suggesting that platelets modulate both the innate and adaptive immune systems. Platelets and platelet-derived microparticles are active in killing foreign pathogens in addition to directing a cascade of proteases and inhibitors to sites of vascular injury and inflammation (70). As well, secreted chemokines and cytokines (RANTES, IL-1β, and MCP-1) and platelet-expressed P-selectin promote leukocyte recruitment, adhesion, and transmigration at sites of vascular injury (Figure 1).

A CD40L-expressing platelet is capable of not only increasing inflammatory responses in the endothelium, but also triggering antigen-presenting cells (dendritic cells and macrophages), resulting in enhanced antigen delivery to T lymphocytes. As demonstrated in CD40L deficient mice infected with viruses, CD40L on activated platelets also facilitates B cell differentiation and Ig class switching (71, 72). Also, patients with immune thrombocytopenia have reduced levels of regulatory T (T_Reg_) cells, and therapeutic increases in platelet counts restore T_Reg_ cell numbers and functions in such individuals (73–75). The effect of TGFβ secreted by platelets in T_Reg_ formation is unclear, but since differentiation of T_Reg_ cells requires TGFβ, platelets may contribute to T_Reg_ formation.

## Platelets in Bacterial and Viral Infections

### In Bacterial Infections

A wide variety of bacteria can interact with platelets, including the *Staphylococci family*, *Neisseria gonorrhea*, *Porphyromonas gingivalis*, and *Helicobacter pylori* (76–78). GPIIb/IIIa, FcγRIIa, and IgG receptors on platelets help them adhere and aggregate around bacteria, as well as fibrinogen and fibronectin (79, 80). TLR1, 2, 4, 6, and 9 within platelets enable them to bind bacteria, which will either cause platelets to secrete thrombocidins (antibacterial proteins within platelet α-granule, including thrombocidin 1 and 2) or aggregate around bacteria to trap them for elimination by phagocytes (20, 81–84). Upon interacting with bacteria, platelets release antimicrobial compounds that are contained within their granules. Alpha toxin released by *S. Aureus* mediates the release of β-defensin from platelets, which is responsible for NET formation (85). Platelets binding to Gram-negative bacteria releases a PF4 with exposed heparin-like epitopes, increasing antibody binding to the surface of bacteria and possibly leading to neutrophil opsonization and phagocytosis (86, 87). Some Gram-negative bacteria, such as *Yersinia pestis*, are insensitive to mammalian TLR4s, making this mechanism crucial (88, 89). Based on *in vivo* experiments with another Gram-negative bacteria, *Porphyromonas gingivalis*, it has been shown that during infection, platelets interact with neutrophils forming heterotypic aggregates in a TLR2-dependant fashion and that TLR2 promotes the aggregation of platelets (90). These study findings indicate that platelets can trigger platelet thrombotic pathway and/or inflammatory pathway activation upon recognizing bacterial components (90).

### In Viral Infections

On the other hand, platelets interact with various types of viruses and their phenotype may vary depending on the type of viral infection (91). Thrombocytopenia and even thrombosis can accompany viral infections (92). Viruses can be divided into those that have either DNA or RNA genomes. Furthermore, RNA viruses can be divided into double-stranded and single-stranded viruses. Several DNA viruses, such as the herpes simplex virus type 1 (HSV1), cytomegalovirus (CMV) and vaccinia, have been identified associated with platelets. However, it is unknown if these viruses can be internalized by platelets (93–95). In contrast, RNA viruses such as HIV, hepatitis C virus (HCV), dengue, influenza, CVB, and EMCV have smaller sizes and are easily internalized by platelets (96–101).

There was recently evidence that SARS-CoV-2, a single-stranded RNA virus, can increase platelet activity and formation of platelet-monocyte aggregates facilitated by TF expression on monocytes (55, 102–105). Koupenova et al. (106) reported that SARS-CoV-2 promotes programmed cell death in platelets in another aspect of platelet-SARS-CoV-2 interaction. According to this study, RNA sequence analysis for SARS-CoV-2 shown by ARTIC v3 sequencing, transmission electron microscopy and immunofluorescence showed that SARS-CoV-2 virions internalized when attached to microparticles. As a consequence of such internalization, apoptosis, necroptosis, and EV release occur, which contribute to impaired immunity and thrombosis (106).

Several studies suggest that platelets may associate with SARS-CoV-2 RNA molecules, and that this event may be more likely to occur in older patients (55, 105, 107). However, Bury et al. did not detect viral RNA in platelets from COVID-19 patients (108). The disparity could be due to the size of the cohort which is significantly larger in the studies that detected traces of viral RNA in platelets of some COVID-19 patients.

## Conclusion

Anucleated megakaryocyte-derived platelets play an important role in hemostasis and thrombosis. Platelets serve as additional mediators of inflammation beyond hemostasis and contribute to several aspects of immune response, including priming of other immune cells and integration of extrinsic immunological stimuli. Indeed, platelets initiate innate immunity as well as adaptive immunity, which is beneficial for host defenses at certain stages of infection. The functions of platelets are thus diversified and rooted firmly in their interactions with vascular and circulating immune cells. Ultimately, uncontrolled endothelial damage and inflammation caused by viral infection progression can result in enhanced platelet reactions which amplify thrombosis and inflammation leading to higher cardiovascular, cerebral and lung pathologic events.

## Author Contributions

YZ and YM wrote and edited the manuscript, read, and agreed to the published version of the manuscript.

## Conflict of Interest

The authors declare that the research was conducted in the absence of any commercial or financial relationships that could be construed as a potential conflict of interest.

## Publisher’s Note

All claims expressed in this article are solely those of the authors and do not necessarily represent those of their affiliated organizations, or those of the publisher, the editors and the reviewers. Any product that may be evaluated in this article, or claim that may be made by its manufacturer, is not guaranteed or endorsed by the publisher.
